# Rodent Hypoxia–Ischemia Models for Cerebral Palsy Research: A Systematic Review

**DOI:** 10.3389/fneur.2016.00057

**Published:** 2016-04-25

**Authors:** Prakasham Rumajogee, Tatiana Bregman, Steven P. Miller, Jerome Y. Yager, Michael G. Fehlings

**Affiliations:** ^1^Division of Genetics and Development, Krembil Research Institute, Toronto Western Hospital, University Health Network, Toronto, ON, Canada; ^2^Department of Pediatrics, Hospital for Sick Children, Toronto, ON, Canada; ^3^Division of Pediatric Neurosciences, Stollery Children’s Hospital, University of Alberta, Edmonton, AB, Canada; ^4^Division of Neurosurgery, Institute of Medical Science, University of Toronto, Toronto, ON, Canada

**Keywords:** spastic hemiplegic cerebral palsy, hypoxia–ischemia, HI rodent model, white matter damage, perinatal brain injury, myelination, oligodendrocyte, periventricular leukomalacia

## Abstract

Cerebral palsy (CP) is a complex multifactorial disorder, affecting approximately 2.5–3/1000 live term births, and up to 22/1000 prematurely born babies. CP results from injury to the developing brain incurred before, during, or after birth. The most common form of this condition, spastic CP, is primarily associated with injury to the cerebral cortex and subcortical white matter as well as the deep gray matter. The major etiological factors of spastic CP are hypoxia/ischemia (HI), occurring during the last third of pregnancy and around birth age. In addition, inflammation has been found to be an important factor contributing to brain injury, especially in term infants. Other factors, including genetics, are gaining importance. The classic Rice–Vannucci HI model (in which 7-day-old rat pups undergo unilateral ligation of the common carotid artery followed by exposure to 8% oxygen hypoxic air) is a model of neonatal stroke that has greatly contributed to CP research. In this model, brain damage resembles that observed in severe CP cases. This model, and its numerous adaptations, allows one to finely tune the injury parameters to mimic, and therefore study, many of the pathophysiological processes and conditions observed in human patients. Investigators can recreate the HI and inflammation, which cause brain damage and subsequent motor and cognitive deficits. This model further enables the examination of potential approaches to achieve neural repair and regeneration. In the present review, we compare and discuss the advantages, limitations, and the translational value for CP research of HI models of perinatal brain injury.

## Introduction: What is Cerebral Palsy?

Cerebral palsy (CP) is the most common pediatric neurodevelopmental physical disability, with a prevalence of 2.0–3.5/1000 births (1). This condition results in a spectrum of motor and developmental disturbances leading to movement disorders, as well as cognitive impairments, speech and language disorders, growth problems, or pain. CP is an umbrella term for several etiologic conditions. Additionally, the term CP describes a range of disturbances in motor function, with spastic CP being the most common condition accounting for 80% of cases (2).

In this review, we will provide an overview of CP, its etiology, and its main pathophysiological mechanisms. Understanding the biological mechanisms of CP has been possible through the use of various animal models, although it is beyond the scope of this review to describe all of them in detail. We will, therefore, focus on rodent hypoxic–ischemic models. Following a summary of the classic hypoxic–ischemic Rice–Vannucci rodent model, we will focus on other HI rodent models developed to mimic the pathophysiological events in humans and discuss the potential of these models in CP research.

Cerebral palsy was originally described by William Little in 1861 (3). Since that time, the definition of CP has constantly evolved alongside scientific and technical progress. In 1997, Palisano et al. described the Gross Motor Function Classification System (GMFCS), which was further revised in 2007. This reference system provides five levels of classification based on self-initiated movements, including sitting, walking, and wheeled mobility (4, 5). In 2006, “The Definition and Classification of Cerebral Palsy, April 2006,” further defined CP:

Cerebral palsy describes a group of permanent disorders of the development of movement and posture, causing activity limitation, that are attributed to non-progressive disturbances that occurred in the developing fetal or infant brain. The motor disorders of cerebral palsy are often accompanied by disturbances of sensation, perception, cognition, communication, and behaviour, by epilepsy, and by secondary musculoskeletal problems.

The definition of CP can be approached from several angles indicating the complexity of the syndrome: (1) the anatomical site of the brain lesion; (2) the clinical symptoms and signs (e.g., spasticity, dyskinesia, or ataxia); (3) the topographical involvement of extremities (e.g., diplegia, quadriplegia, or hemiplegia); (4) the timing of presumed insult (e.g., prepartum, intrapartum, or post-neonatal); and (4) classification of the degree of muscle tone (e.g., isotonic, hypotonic, or hypertonic) (6, 7). CP patients often present with mixed symptoms. For research and knowledge transfer purposes, a standardized and more consistent classification has been proposed (1, 8). However, no simple definition of CP exists; this issue is discussed further in an excellent review by Rosenbaum and colleagues (9).

## Etiology of Cerebral Palsy

**Figure 1:**
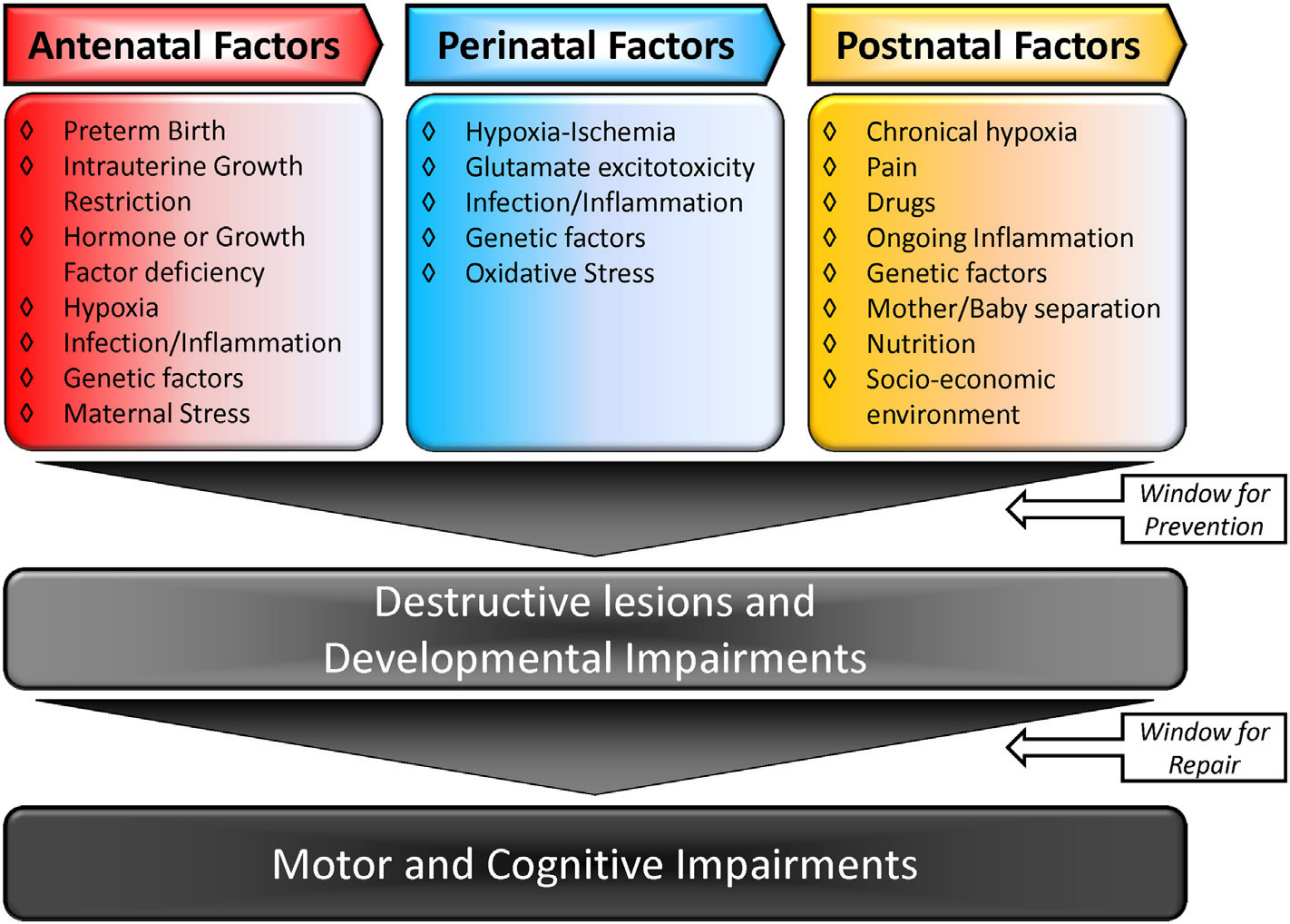
**Multiple-hit hypothesis of cerebral palsy: a combination of two or more factors is more likely to trigger and modulate lesions in the brain, causing CP**. Adapted from Ref. (10, 15).

**Figure 2:**
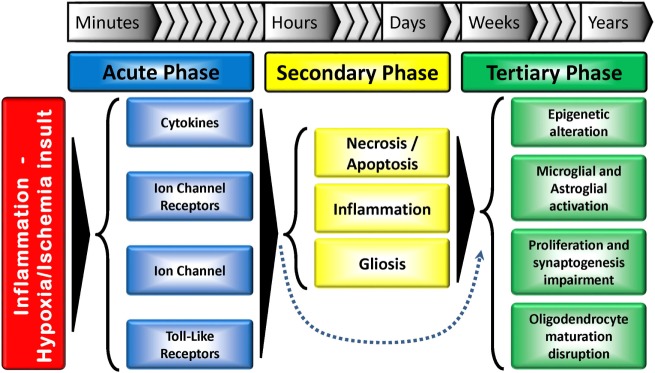
**Tri-phasic hypothesis of the perinatal brain damage: major action elements and processes**. The acute phase occurs minutes after the insult, while the secondary phase happens hours after. A tertiary phase, which can last for months/years and result in developmental disturbances has also been proposed. The secondary phase can be skipped in some situations. Adapted from Ref. (15).

The causal factors for CP are multiple and may be linked. Figure 1 summarizes the multiple-hit hypothesis of CP pathobiology. CP can follow a brain abnormality that occurs pre, peri, or postnatally or some combination thereof. Even when the injury occurs at a defined time, moderating factors may also exist (10, 11). The risk factors for CP include: preterm birth, intra-uterine growth restriction, maternal/fetal infection, inflammation, perinatal and intrapartum difficulties (e.g., hypoxic–ischemic insult), and genetic predispositions (1, 12, 13). Remarkably, intrapartum hypoxia–ischemia (HI) is thought to account for only 14.5% of CP cases (14). The importance of inflammation in CP onset has also been recently reviewed by Van Steenwinckel and colleagues [Figure 2; (15)].

A recent study has shown *de novo* copy number variations (CNVs) in the genome are clinically relevant to CP (16). Furthermore, genes, such as *KAK1*, *AP4MI*, or *GAD1*, have been shown to play a role in up to 45% of CP cases (17, 18).

It has been suggested that males and females may also respond differently to developmental brain injury. For instance, males born preterm (at 28 weeks of gestation in these studies) have been reported to be more susceptible to white matter damage (19, 20).

Developmental stage also plays a crucial role in determining the response to an insult. Generally, insults occurring during the first trimester of gestation are linked to abnormal cerebral development (e.g., schizencephaly). Insults in the late second and early third trimester typically lead to periventricular white matter injury (PVWMI), often associated with neuronal abnormalities but not necessarily neuronal loss, while insults in the late third trimester trigger cortical and deep gray matter lesions (21). Up to 24 weeks of gestation, cortical neurogenesis is defined by proliferation, migration, and formation of neural precursor cells and neurons. In the following weeks of gestation, other dynamic mechanisms take place: growth (axon/dendrites formation), differentiation (synaptogenesis, myelination), and maintenance (i.e., apoptosis, pruning) and/or specialization (development of circuitry). These mechanisms persist up to 2 years after birth (10, 22). Therefore, any injury occurring in the immature brain will trigger a cascade of developmental disturbances (Figure 2).

## Pathophysiology

### Characteristics of Brain Lesions: Periventricular Leukomalacia

One of the most common pathological causes of CP is periventricular leukomalacia (PVL). PVL has three forms: cystic, non-cystic, and diffuse. Cystic and non-cystic forms of PVL encompass aspects of focal necrosis and diffuse injury of the white matter. While cystic focal necrosis, which is macroscopic, turns into cysts, the microscopic non-cystic necrosis turns into glial scars (23, 24). A diffuse white matter injury is characterized by astrogliosis and microgliosis as well as hypo-myelination, due to lower amounts of pre-myelinating oligodendrocytes (OLs) (22, 24, 25). These lesions can be accompanied by hemorrhage in the germinal matrix, the periventricular zone, or into the ventricle (26–28).

There are a number of potential underlying mechanisms for diffuse PVL. The most studied mechanism is pre-oligodendrocyte (pre-OL) injury: a disruption of the OL lineage leading to serious impairment of axonal myelination and development caused by a deficit of trophic factors release (29, 30). Also, as a secondary event, axonal injury has been proposed as a mechanism leading to axonal degeneration, hypo-myelination, and decreased cortical and thalamic volumes. The subplate neurons, which are among the first neurons to appear in the cerebral cortex, play a crucial role in the development of the cerebral cortex and deep nuclei (wiring and functional maturation), and their impairment triggers secondary and maturational effects. Neonatal HI causes selective subplate neuronal death, which is responsible for abnormal cortical development (31). Subplate neuronal injury subsequently leads to plasticity impairments (32, 33). Thalamic development is also affected. In fact, thalamic neuronal injury leads to white matter axon damage and gliosis. Finally, the dorsal telencephalic subventricular zone and the late GABAergic migrating neurons, which are crucial to the structure of the upper cortical layers, are prone to primary injury [see Ref. (27)].

Davatzikos et al. report that the growth of the corpus callosum, which connects the left and right cerebral hemispheres and contains commissural myelinated fibers, is severely impaired in premature infants with PVL, though type of PVL is not specified in this work (34). This pathology preferentially occurs around the lateral ventricles, which are close to major cerebral arteries. This also affects descending corticospinal tracts (CSTs) and leads to neuromotor deficits, including CP (34–36). Subplate neurons have a fundamental role in the elaboration of connections between the thalamus and the cortex, as well as within the cortex (37). Thus, thalamocortical, corticocortical, and corticospinal synaptogenesis are impaired, leading to compromised connectivity and mapping in rodents (2, 38, 39). The most prominent anatomical feature in the onset of spastic CP is the lesion of the sensorimotor cortex and its subcortical white matter (40).

It has generally been understood that there are primary and secondary injury events in PVL. The primary event is reported as being a key contributor to the pathobiology while, in the secondary phase, developmental disturbances involving the cerebral white matter and other structures: thalamus, basal ganglia, cerebral cortex, brainstem, and cerebellum occur (27, 41). Interestingly, recent studies have shown that growth impairments are not linked to neuronal death, but rather to dendritic arbor maturation alteration, spine density dendritic network decrease (42), and disturbance in synapse formation of the cortical neurons (43).

Recently, a tertiary phase of persisting injury, lasting for months or years, linked to persistent inflammation and possibly epigenetic changes, has been identified (Figure 2). These long-term epigenetic modifications, which have been suggested as potential mechanisms involved in various pathologies, include phosphorylation, ubiquitination and acetylation of histones, and methylation of DNA and RNA. This third phase not only inhibits regeneration and/or exacerbates brain damage, but also sensitizes the brain to additional injury, such as myelin deficits. Cerebral plasticity is also reduced, further contributing to the abnormal development of the brain (20).

### Disruption of Oligodendrocyte Maturation

In humans, susceptibility to PVWMI peaks between 23/24 and 32 weeks of gestation (44, 45) This vulnerability to hypoxia/ischemia/trauma has several causes, including the higher energy needs of the brain to support the rapid development of white matter. Also, as the brain grows, the distance between blood vessels increases, which makes the oxygen and blood supply more critical (46, 47). It is now apparent that OL precursor cell intrinsic hypoxia-inducible factors (HIFs) signaling is a key coupler of white matter angiogenesis and the onset of myelination postnatally (48).

Myelination impairment is linked to the disrupted OL differentiation process, characterized by a deficit of myelinating mature OL (22, 49, 50). Back et al. showed that PVL has a developmental window that starts before the beginning of myelination, when late OL progenitors are the predominant OL type. When myelination starts in the periventricular white matter, a decline of PVL incidence is also observed. This indicates that the presence of late OL progenitors (Pre-OLs) is linked to the risk of PVL and defines them as a preferential target in PVL (44, 51).

Comparative timetables between human and other species for the various developmental stages have been proposed to facilitate the understanding of PVWMI onset during development. The differences of brain structure and development between human and rodents do not allow perfect comparisons. However, Table 1 summarizes the human and rodent OL developmental stages. Briefly, embryonic day (ED) 17 to postnatal day (PND) 2 in rodents corresponds to 14–23 gestation weeks in humans. PNDs 2–3 in rodents are similar to 23–32 weeks in humans (preterm infant). PNDs 7–10 in rodents are comparable to 36–40 weeks in humans (term infant). A rodent at PNDs 20–21 is comparable to a 2–3 years old human (2, 45, 52–54). Contradictory opinions exist regarding the timing of maturation of OLs. In a review by Semple et al., it was suggested that the maturation peak of Pre-OLs occurs at PNDs 1–3 (45), by contrast, a time window of PNDs 2–5 has been suggested in a review by Clowry (2, 55). However, several reports indicate that the maturation peak occurs at PND 2 (56–58). Moreover, Dean et al. showed that immature OLs peak at PND 5 in rat, while Semple et al. suggested a predominance of immature OLs between PNDs7 and 10 (45, 58). It is now clear that OL progenitors persist into adulthood, contributing to myelination processes as well as to other mechanisms, such as neuronal activity modulation (59, 60).

**Table 1:** **Comparative timetable of oligodendrocyte predominating subtypes during development in human and rodent**.

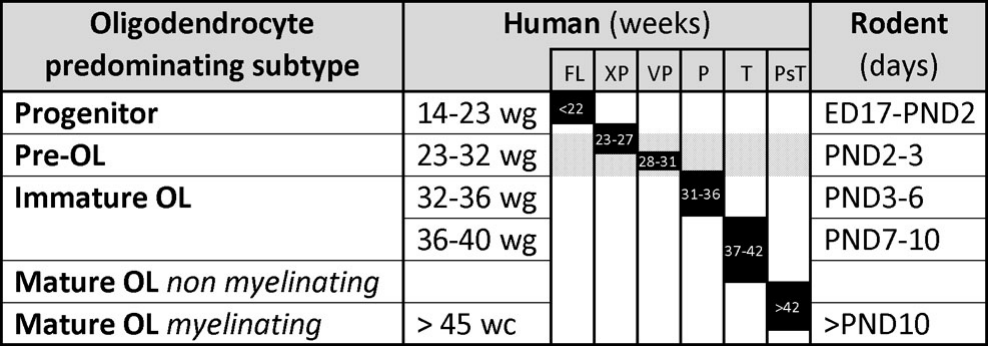

*This table is an indication of the accumulated literature, which includes divergent publications. We do not intend to suggest that the subtypes exist only within these time windows. OL, oligodendrocyte; wg, weeks of gestation; wc, weeks after conception; ED, embryonic day; PND, PostNatal Day; FL, fetal loss; XP, extremely preterm; VP, very preterm; P, preterm; T, term; PsT, post-term. Adapted from Ref. (2, 45, 56, 58, 61)*.

*Period of highest susceptibility to periventricular white matter injury 
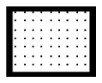
*.

### Cellular Mechanisms

Ischemia/oxygen glucose deprivation and inflammation are the main mechanisms that initiate PVL and they can facilitate each other (56, 62). Increase of pro-inflammatory cytokines has also been linked to PVL (63, 64). Interestingly, models of ischemic brain injury, such as the intra-uterine growth restriction model show damage that mimics CP conditions (65–67).

**Figure 3:**
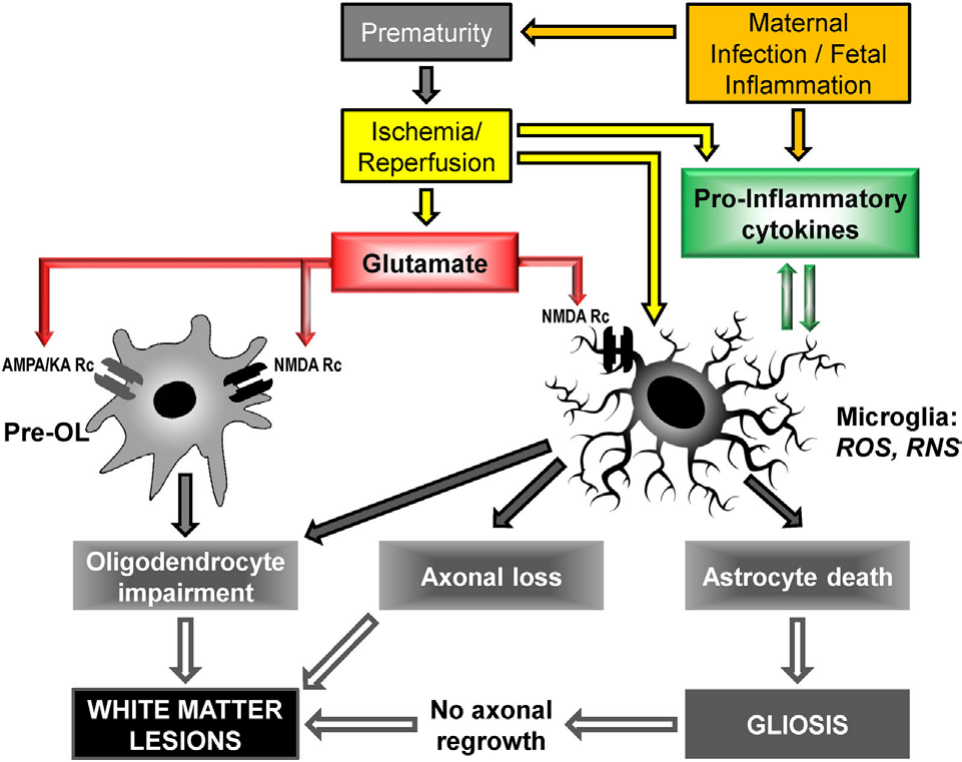
**Cellular and molecular mechanisms to PVL onset: upstream mechanisms (cerebral hypoxia/ischemia and maternal infection/fetal inflammation) activate downstream mechanisms (glutamate excitotoxicity and free radical release) leading to cellular deficiencies, in particular oligodendrocyte maturation impairment and myelination deficit**. Pre-OL, pre-oligodendrocyte; Rc, receptor; AMPA, α-3-amino-hydroxy-5-methyl-4-isoxazole propionic acid; KA, kainate; NMDA, N-methyl-D-aspartate; ROS, reactive oxygen species; RNS, reactive nitrogen species [adapted from Ref. (23, 47, 50, 68, 69)].

Other mechanisms include neurotransmitter-mediated excitotoxicity and oxidative stress (free-radical release), as well as alteration of the extracellular matrix (23, 47). Figure 3 shows the cellular and molecular mechanisms triggered by glutamate and pro-inflammatory cytokines.

Pre-OLs, more so than immature OLs, are particularly vulnerable to HI insults. Early OL progenitors respond to white matter injury by a rapid and significant phase of proliferation. The pool of pre-OLs is, thus, dramatically increased. However, these cells fail to differentiate further and do not repair white matter (50, 53), resulting in development arrest that is likely linked to inhibitory factors released by reactive astrocytes, such as interleukin-1, and microglia (15, 49).

Oligodendrocytes express neurotransmitter receptors [e.g., glutamate and adenosine-triphosphate (ATP)] and respond to signals conveyed by axons. Under pathological conditions, they trigger a surge of intracellular calcium that disrupts the Ca^2+^ homeostasis to which OLs are highly sensitive (70).

Hypoxia–Ischemia caused by oxygen and glucose deficits leads to a decrease in ATP which in turn compromises the sodium–potassium ATP pump, crucial for membrane polarity maintenance (69). Interestingly, during ischemia, anoxic depolarization initiates extracellular ATP release from glial cells (71).

Adenosine-triphosphate-mediated toxicity, through the ionotropic P2X and the metabotropic P2Y purino-receptors (72), leads to apoptosis or necrosis and is correlated with the degree of intracellular Ca^2+^ surge (73).

Depolarization induces excessive glutamate release, which activates the ionotropic glutamate receptors AMPA, Kainate, and NMDA present in OLs. In addition, activation of metabotropic glutamate receptors (mGluRs), strongly expressed by pre-OLs, leads to a calcium influx (74, 75). Interestingly, the reuptake function of the glutamate transporter (GluT) is disrupted in pathological conditions after HI and the glutamate is released instead of being recaptured, which further increases its extracellular concentration (76).

Calcium is responsible for the activation of diverse enzymes, such as phospholipases, proteases, endonucleases, and neuronal nitric oxide synthase. The surge of intracellular calcium affects the mitochondria, which captures cations and subsequently produces free radicals and reactive oxygen/nitrogen species (ROS/RNS). Oxidative stress has a profound impact on enzyme function, transcription factor expression, lipid oxidation, and DNA damage (47, 69).

Various other neurotransmitter receptors, such as Dopamine D2 and D3, GABA_A_, and Adenosine A receptors have been shown to play a role in mature OL injury after HI (75, 77, 78). Other mechanisms include upregulation of the expression of members of the kinin protein family. OLs respond to kinins via specific receptors that elicit a calcium influx (79).

The presence of a systemic inflammatory state sensitizes the brain to injury. Increased concentrations of peripheral cytokines are a marker of cerebral inflammation since, in a neuro-inflammatory context, a sustained release of cytokines alters the blood–brain barrier. Infection can predispose the fetus to white matter lesions, leading to neurological impairments (15). Interestingly, some studies failed to show a direct causal link between chorioamnionitis or fetal infection and early brain development abnormalities in preterm infants, while neonatal infection shows higher risk of injury (80, 81). However, several other animal studies have shown a strong link between fetal infection and brain injury. For example, intraperitoneal injection of interleukin-1β (IL-1β) in newborn mice triggers systemic inflammation that modifies programs controlling brain development (82). IL-6 has been shown to be a key trophic factor for OL survival *in vitro*, while this effect is increased when associated with other trophic factors, such as neurotrophins (83). IL-6 and tumor necrosis factor α stimulate astrocyte proliferation and inhibit neural precursor cell proliferation in rodents. Lipopolysaccharide (LPS) has also been shown to cause susceptibility to hypoxic–ischemic insult in the newborn brain (84). Remarkably, while cytokines can be pro-inflammatory and act as mediators in the infection process, they also can have neuroprotective properties (63).

Inflammation can also trigger gliosis, which is a non-specific response to injury from glial cells, such as astrocytes and microglia. Microglia are among the first cells to react to injury and mediate neurotoxicity and they are strongly involved in the inflammatory response (85). Reactive astrocytes contribute to the glial scar and can subsequently inhibit axonal regrowth and myelination (86). Inflammation also contributes to microglia activation and augments their phagocytic capacity (20), leading, for example, to phagocytic degeneration of axons and necrotic/apoptotic cells (87). Microglia and reactive astrocytes also release interferon γ, which has a damaging effect on pre-OLs in PVL (88).

Other components, altered by the inflammation process, play an important role in glial scarring. For example, chondroitin sulfate proteoglycans (CSPGs) and immunoglobulin cell adhesion molecules (IgCAMs) inhibit regeneration, and activation of toll-like receptors (TLRs) alters the neuroimmune response and OL maturation (20, 89, 90).

A recent study, however, suggested that hyperoxia (excess of oxygen) and/or hypoxia can alter the expression of various genes, independent of any oxidative stress mechanisms (24). The level of oxygen can regulate transcription factors, such as the HIFs, which are heterodimers of an oxygen-regulated α subunit and an oxygen-insensitive β subunit. Three HIFs have been described to date. In the absence of oxygen, HIF1 and 2 are deactivated by asparaginyl and prolyl hydroxylases, two oxygen-dependent enzymes. Thus, HIFs cannot form a heterodimer with the β subunit and translocate to the nucleus to elicit specific DNA transcription (91). Therefore, postnatal myelination (starting at PND7–9 and peaking at PND15–21) is impaired if pre-OLs-encoded HIF function is altered by the absence of oxygen (48).

In summary, CP is triggered during a time of rapid brain development and maturation with many mechanisms at play, most notably an alteration of brain development. The major pathophysiological events occurring during CP onset in both rodents and humans may be seen in Table 2.

**Table 2:** **Major pathophysiological processes in CP reproduced in the rodent HI model**.

Pathophysiological processes	Human patients with CP	HI model
**Structural injury**		
Brain injury in the ipsilateral hemisphere, from slight to severe, in the cortex, striatum, hippocampus, and corpus callosum. Infarctions observed in the cortex and hippocampus	(27, 41, 92)	(93)
Focal injury (cystic and non-cystic PVL) and diffuse injury (astrogliosis, microgliosis, oligodendrocyte death) in the white matter, cortex, and subcortical structures. Hemorrhages	(10, 27, 92)	(27, 93)
**Cellular injury**		
Selective vulnerability of late oligodendrocyte progenitors to hypoxia–ischemia: rodent postnatal days 2–3; human gestational weeks 23–32	(2, 44, 45)	(2, 45, 56)
Oxidative stress: free radical release	(10, 62, 94, 95)	(23, 96, 97)
Glutamate-mediated excitotoxity: involvement of NMDA and mostly AMPA receptors		(68, 69, 96, 98)
**Metabolic disturbances**		
Blood flow decrease in white matter and subcortical white matter structures	(99, 100)	(101)
Hyperventilation, hypocapnea, metabolic compensation	(92)	(102)
Glucose and oxygen exhaustion in the ipsilateral hemisphere		(103)
Retardation of anaerobic glycolytic flux, ATP depletion, and ultimate tissue damage		(101)
**Role of inflammation**		
Contribution of perinatal infection to the evolution of the damage	(15, 104, 105)	(82, 84, 106)
**Genetic component**		
Hypoxia–ischemia has an impact on gene expression	N/A	(24, 48)
Genetic predisposition	(16–18, 107)	(19)

## Cognitive Impairments

In addition to motor impairment, as categorized by the GMFCS, children with CP are more susceptible to several other health problems. These include orthopedic problems, epilepsy, chronic pain, as well as sensory and cognitive impairments. Indeed, children with CP often exhibit cognitive and behavioral deficits, including lower IQ, deficits in short-term memory, and hearing impairment frequently resulting in speech deficiencies and/or delayed language development (108, 109). Moreover, visual perception is often affected by CP, and is considered to be a core disorder of the CP syndrome (110).

Very preterm or very low birth weight infants are at significant risk of developing cognitive impairments (111). Neonatal brain injury in these children leads to adverse attention and processing speed outcomes at the age of 7 (112). MRI has become a tool to assess neonatal brain injury and predict potential childhood outcomes (113, 114).

Animal studies have helped to characterize these cognitive impairments. Neuro-behavioral tests in animal models of CP have shown a number of cognitive impairments, including selective and long-lasting learning and memory impairments (115), and deficits in auditory processing (116).

Interestingly, male HI rats can learn a complex spatial memory task if exposed to gradual and repetitive situations, whereas performance is reduced if a high memory demand is requested at once (117). Furthermore, differing results have been observed according to which brain hemisphere is injured, supporting the notion that the neonatal right brain is more vulnerable to HI (118, 119). However, many human studies and animal bilateral injury models have failed to confirm the concept of brain lateralization, suggesting the need for further investigation in this area.

## Potential Therapies

Therapeutic hypothermia is a standard of care procedure for term infants with moderate or severe perinatal asphyxia (120–122). This treatment is, however, not recommended in preterm infants due to potential collateral damage, such as intra-cerebral hemorrhage (122).

In an immature newborn, therapeutic strategies could interfere with the normal development of the brain making use of potential neuroprotective agents risky. Robertson et al. reviewed several compounds, including US-Food and Drug Administration (FDA) approved (i.e., Tetrahydrobiopterin, Melatonin, Erythropoietin) and not FDA approved (i.e., Xenon, Epo-mimetics), that could be used to treat the newborn infant (123).

Fleiss and colleagues (20) also reviewed the treatment options for tertiary brain damage, including targeting long-lasting inflammation, modulation of microglia or lymphocytes, and cell therapy.

The use of stem cells as a potential regenerative approach seems to be a promising strategy. Functional recovery after stem cell transplantation has been shown in many cases, including in spinal cord injury models (124, 125) and in a dysmyelinated animal model (126). However, many aspects of cell therapies, such as safety, efficacy, timing, and dose, still need to be optimized (127), while a combinatorial approach with, for example, rehabilitative strategies, use of growth factors or engineered stem cells, is likely to be the optimal strategy rather than any of these alone (128).

Clinical trials for CP encounter difficulties defining recruitment criteria and recruiting participants, mainly due to the heterogeneous patient population or inadequate group sizes. Rodent models can be of great help since they allow the examination of biological mechanisms in a safe and cost-effective manner. They are also a useful tool for testing therapeutic strategies. For example, specific microRNAs have been shown to regulate T-cell function and intracellular ATP concentrations. Furthermore, growth factors as well as drug and cell therapies are being investigated in animal models (20, 129).

Therefore, the need for rodent models of CP is great. However, each adaptation of the classic hypoxic–ischemic Rice–Vannucci model should be proven to be clinically relevant, and used appropriately once validated. The following sections discuss the advantages, limitations, and translational value of these models.

## The Classic Rice–Vannucci Model

The Rice–Vannucci rat HI model (93) is the first and best studied HI neonatal model. This model allowed the study of the mechanisms underlying HI-related brain damage during development and is one of the most productive models of perinatal brain injury to have been described (130).

This model, using postnatal day 7 (PND7) neonate rats, is an adaptation of the Levine model of HI in adult rats. It is widely accepted that the 7-day-old rat has brain maturity equivalent to that of a term/near term human fetus (131).

Briefly, this model is accomplished as follows: the common carotid artery is ligated unilaterally in PND7 Sprague-Dawley rat pups. The animals rest for 4–8 h before being exposed to hypoxic air (8% oxygen) for 3.5 h at 37°C. They are subsequently returned to their dam. In this original publication, brain damage was observed after 50 h in the ipsilateral hemisphere but not in the contralateral one (93). Others have observed damage much earlier (e.g., 2 h following the insult) (132). Using these experimental conditions, hypoxia alone (without carotid occlusion) does not result in brain damage nor does carotid unilateral occlusion alone (without hypoxia) (133). After unilateral carotid occlusion alone, a regulation of the ischemic effect has been observed due to the presence of the circle of Willis that allows compensation in the blood flow between the brain hemispheres.

The injury induced in this model can be described as a spectrum, ranging from “moderate” to “severe.” Selective neuronal death or infarction can be observed, depending on the severity of the HI insult (93, 133, 134). The injuries affect the cerebral cortex, the deep gray matter (striatum of the basal ganglia and thalamus), the subcortical and periventricular white matter, and the hippocampus in the majority of the animals (90%). It should be noted that only 56% of the animals have an infarction, suggesting a variability in the technique.

A hypoxic–ischemic insult of an inadequate level to induce rapid cell death and infarction can trigger some prolonged injury mechanisms. Glutamate excitotoxicity as well as oxidative stress play crucial roles in cell death following HI insult (133).

This model has been adapted many times since it was originally described. It has been confirmed to be extremely useful in various studies of perinatal hypoxic–ischemic brain damage. Since its first description in 1981, the Rice–Vannucci model has been widely used and adapted by scientists around the world, on various species.

However, studies have revealed that the model does not mimic the human condition optimally. Specifically, the stage of white matter development in PND7 rats and the type of injury produced are not similar to that in humans. At PND7, the majority of OLs in rat brains are at the immature stage of development as defined by O4+/O1+ immunohistochemistry (57). By contrast, human injury occurs when OLs are at the earlier stage of development, which can be distinguished by O4+/O1− staining (44). O4+/O1− staining shows that the corresponding period in rodents is PND 2 (56–58). Another important consideration involves the type of injury the model aims to reproduce. Most cases of PVL resulting in CP are associated with diffuse apoptotic and relatively small necrotic areas in the infant brain, affecting mostly white matter (23, 92, 135), while the Rice–Vannucci model often results in severe injury with multiple infarctions involving both white matter and gray matter (93, 136), which accounts less than for 5% of PVL cases (137–139).

## Subtypes of the Rodent HI Model

In recent years, several optimizations of the rodent HI model have been developed to match the pathophysiological processes underlying brain injury and the consequences observed in human CP patients. Major differences in brain anatomy and the speed of brain development exist between humans and rodents. Fine-tuning of the timing and the parameters of the injury insult have resulted in a more accurate reproduction of the changes observed in humans (Table 3).

**Table 3:** **Hypoxia–ischemia (HI) and ischemia^a^ rodent models developed to mimic pathophysiological processes and conditions in infants with prenatal asphyxia**.

Species, Reference	Insult	Age at the time of injury/duration of the surgery	Age at the time of result acquisition	Anatomical injury	Immune/inflammatory response	Functional injury	Contribution/advantage of the model
Sprague-Dawley rats (93)	Left common carotid artery ligation, halothane anesthesia, 8% O_2_ for 3.5 h at +36°C	P7/not exceed 20 min	P9	From slight to severe injury found in the cortex, striatum, hippocampus in ipsilateral hemisphere, and corpus callosum in 90% of animals. Infarctions in 56% of animals, involving cortex and hippocampus	Activated microglia, astrocytes, fibroblast-like elements in the adventitia of arteries. Activated macrophages were identified around infarction zones	No neurological abnormalities (no circling, hemiparesis, convulsions), normal reflexes	Brain edema is a consequence, not a cause, of brain damage. Heightened vulnerability of the foci of myelinogenesis similar to humans. The first and the best studied HI model
Sprague-Dawley rats (140)	Right common carotid artery ligation, halothane anesthesia, 6% O_2_ for 3.5 h at +37°C	P1	P2–P10	Severe unilateral injury. Necrosis in the cortex, thinning of anterior corpus callosum	Microglia activation from days 2 to 6, progressive neuronal loss in the cortex and astrogliosis in the cortex, hippocampus, and corpus callosum	Not reported	The first HI model to study the effect of earlier HI injury (at P1) which corresponds to prenatal period in humans. Evolution of brain damage following HI injury
C57Bl/6, 129Sv and CD1 mice (141)	Right common carotid artery ligation, halothane anesthesia, 8% O_2_ for 30, 60, and 90 min at +37°C	P7	Five days after the HI procedure	CD1 strain was the most susceptible for the injury, and induced the least mortality. 129Sv appeared to be the most resistant strain with high mortality. CB57 – intermediate vulnerability with high mortality	Not reported	Not reported	Comparison of HI injury effects on different mouse strains: CD1 and CD1 × CB57 hybrids are the best mouse strains to study HI injury mechanisms
Wistar rats (142)	Bilateral common carotid arteries occlusion (cut) (BCAO model), sodium pentobarbital anesthesia, no hypoxic conditions, +36–37°C for recovery	P5	P7	Ninety-one percent of animals showed mild to severe white matter lesions in corpus callosum internal capsule, subcortical white matter (HE staining, gradual scoring). Cortical neurons were spared (APP marking)	Not reported	Not reported	First model of permanent bilateral artery ligation. Coagulation necrosis, cystic, and diffuse lesions, selectively in white matter
Wistar rats (143)	Bilateral common carotid artery *temporary* ligation, for 10 min, during which pups were kept in 8% O_2_	P7	Six and 24 h after HI procedure	Significant depletion of oligodendrocytes (O4 +) in corpus callosum, at both 6 and 24 h after injury. Astrocyte counts appeared normal (GFAP)	Not reported	Not reported	First HI model to investigate *temporary* effects of hypoxic–ischemic conditions on cell survival
Long-Evans rats (96)	Unilateral common carotid artery ligation, anesthesia with ether, hypoxia 6% O_2_ for 1 h at +33–34°C	P7	48–96 h after HI	Selective subcortical white matter injury (MBP expression loss, ISEL-positive cells exclusively in the white matter). AMPA receptors were at maximal concentration at P7 compared with P4; AMPA antagonist attenuated injury maximally at P7, compared to P4 and P11	Not reported	Not reported	Model of selective white matter injury, which was found to be mediated by AMPA receptors at P7
Sprague-Dawley rats (144)	Bilateral common carotid arteries ligation, pups kept at +37°C for recovery. Isoflurane anesthesia. No subsequent hypoxic conditions	P1	P7 and P14	Decreased number of immature (O4+/O1+) and rare mature (MBP+) oligodendrocytes at P7	At P7, activation of macrophages and microglia (ED1, OX42)	Not reported	Preferential diffuse injury of subcortical white matter, without coagulation necrosis and cystic lesions
Wistar rats (84)	Unilateral right carotid artery cut, anesthesia agent not mentioned, 7.7% O_2_ for 10–50 min. Four hours before HI procedure, a single dose of lipopolysaccharide (LPS) was given, 0.3 mg/kg i.p.	P7	P10	LPS administration in combination with HI procedure induced much more severe tissue injury (area of infarction, astrogliosis, microglia presence) compared to HI alone	CD14 mRNA expression significantly increased both following LPS/HI and LPS alone. Toll-like-receptor (TLR)-4 mRNA was decreased in LPS/HI group	Not reported	Study has established a model to study contribution of perinatal infection to the evolution of brain damage
Sprague-Dawley rats (56)	Left common carotid artery ligation, anesthesia method not mentioned, 6% O_2_ for 4 h at +37°C at P2 or 8% O_2_ for 2.5 h at P7	P2 and P7	Twenty-four hours and 1 week after HI injury	Following HI at P2, late OL progenitors were decreased (O4+/O1−), and several markers of cell death were increased (cytochrome c accumulation in cytoplasm, caspase-3 activation, TUNEL). At P7, 92% of OLs were at immature stage (O4+/O1+), and were much less vulnerable to HI insult than late OLs progenitors (O4+/O1−)	Not reported	Not reported	Selective vulnerability of late OL progenitors to hypoxia–ischemia
Sprague-Dawley rats (145)	Left common carotid artery coagulation, halothane anesthesia, 10 min of hypoxia with 6% O_2_ at +35–37°C	P3/10 min	Three days after HI	Significant neuronal damage was found in cortical regions in the brain that are associated with sensorimotor integration and movement control, thalamus and basal ganglia, but not in hippocampus (fuchsin–thionin staining). Apoptotic and necrotic cells were described (TUNEL), as well as astrogliosis (GFAP) in the above areas. White matter damage was not quantified	Not reported	Not reported	Consistent pattern of mild cortical injury seen in a surviving preterm human infant. White matter damage was not quantified
Sprague-Dawley rats (146)	Bilateral carotid artery ligation, isoflurane anesthesia, HI 8% O_2_ for 10 or 15 min	P4	P21	Severe (15 min of hypoxia) brain injury: decreased thickness of corpus callosum at bregma level (LFB), and the number of mature oligodendrocytes (APC staining). HI of 10 min has not induced significant alterations	Not reported	Righting reflex, wire hanging test, cliff avoidance test, and locomotor activity did not reveal any differences. Several gait parameters were impaired, increased preference of open arms in a plus-maze test	Fifteen minutes of hypoxia-induced severe damage to white and gray matter with identified necrotic cells; several behavioral impairments were found
Wistar rats (147)	Right common carotid artery cut by thermocauterization, isoflurane anesthesia, HI 8% O_2_ for 60 min at +37°C	P3/5–10 min	24–48 h and 6 weeks after HI	Mild myelin loss (MBP staining) and cortical damage was observed 6 weeks after injury in both genders. Females only: 2-iminobiotin (2-IB) prevented release of cytochrome *c* from mitochondria (caspase-3-dependent pathway of apoptosis) (24 and 48 h after HI), and brain damage (6 weeks after HI)	Not reported	Not reported	Pathways leading to HI-induced cell death are gender-dependent: in females, capase-3-mediated apoptosis, but not in males
C57Bl/6 mice (148)	Unilateral left carotid artery ligation, isoflurane anesthesia, 10% O_2_ for 50–80 min	P5	P12	HI for 70 min induced white (MBP staining) and gray matter injury (MAP2), including hippocampal atrophy. 50 min of HI injured a small percentage of animals and no white matter damage (MBP staining), 80 min-extensive infarction in multiple areas	HI 70 min: primarily Th1/Th17-type immune response: upregulation of T-bet, IL-6, 22, 12a. Increased expression of TREM2 and DAP2 in white and gray matter (innate immune response)	Not reported	Seventy minutes of HI produced white and gray matter injury resembling preterm brain injury. Innate and adaptive immune responses are involved, with strong bias to Th1/Th17. TREM2 and DAP2 are implicated in oligodendrocyte pathology

*^a^Ischemia models refer to some bilateral carotid artery occlusion (BCAO) models that did not use hypoxic conditions following artery ligation/cut. Analyzed here for the purpose of comparison as closely related models*.

The first optimization of the HI model was done in 1996, when hypoxic–ischemic injury was induced in rat pups at PND1 (140). At PND 1, rat brain development is at a comparable stage to the human brain in the last trimester of gestation (149). Sheldon et al. showed that evolution of brain damage following HI includes progressive neuronal loss, white matter thinning, activation of microglia, and macrophages (140). Rat brains with this injury present with infarctions, necrotic areas, and cystic lesions similar to those seen in infants with severe forms of PVL (28).

As more research was undertaken using the HI model, concerns about strain differences were raised. In particular, susceptibility to the injury, mortality rates, and post-injury plasticity. Studies were commenced to obtain reproducible HI rodent models with controllable and standardized degrees of brain damage. Different strains of mice were compared in a study by Sheldon and colleagues (141), in which the CD1 strain was found to be preferable as it showed maximum brain vulnerability with little or no mortality. 129Sv and CB57 strains were found to be extremely resistant to injury and displayed very high mortality, respectively.

One year later, in 1999, a new HI model was developed which employed bilateral common carotid artery occlusion (BCAO) in Wistar rats at PND5 with no subsequent hypoxic conditions (142). The authors aimed to reproduce the ischemic conditions that occur in the human fetus, i.e., reduction of cerebrospinal fluid (CSF) flow resulting in mostly white matter lesions. In the Rice–Vannucci model, CSF flow in subcortical white matter was reduced by 60% (101), and was followed by severe white and gray matter damage. By contrast, in the BCAO model, CSF flow in subcortical white matter was reduced by 25%, which resulted in mild diffuse white matter damage in 64% of brains, or severe white matter damage with necrotic areas and cystic lesions in 36% of brains, while gray matter was spared. However, other researchers have failed to reproduce this model and the contradictory data warrant further investigation.

Also in 1999, another BCAO model was developed, which studied the effect of *temporary* bilateral ligation of common carotid arteries at PND7 in rats (143). Ligation was carried out for 10 min during which rat pups were kept in 8% oxygen. Progressive loss of O4 marked OLs was observed 24 h after injury. The report is very short and no other data were available, but this BCAO model is likely to produce more severe brain damage than the BCAO model by Uehara, since hypoxic conditions were added to the ischemic injury.

A study by Follett and colleagues also attempted to generate a reliable model of white matter injury (96). Parameters of the insult were changed in order to produce less severe brain damage: instead of 8% oxygen and 3.5 h of hypoxia at +37°C used in the Rice–Vannucci model, unilateral carotid artery occlusion was followed by 6% oxygen for 1 h at +33–34°C. Mild hypothermia during hypoxia has been shown to significantly reduce brain damage in rat pups when operated on at PND7 (150), and this was confirmed here. Selective mild white matter damage was seen 24–96 h after the HI procedure, as demonstrated by MBP expression loss and exclusive white matter ISEL (*in situ* end labeling) staining. The authors found that OL death was associated with activation of glutamate AMPA receptors, and vulnerability to AMPA activation was maximal at PND7 as compared to PND4 and PND11. At PND7, the number of AMPA receptors on OLs (most at the immature stage of development, O1+ marked) is significantly greater than that at PND4 [when pre-OLs (O4+/O1−) predominate], or at PND11 (mature cells, MBP-positive). Administration of the AMPA blocker NBQX prevented brain damage in animals at PND7, but not at PND4 and PND11. In humans, increased numbers of AMPA receptors were also found in pre-OLs and immature OLs between 24 and 32 weeks of gestation, and the mechanisms of glutamate-mediated cell death look similar (151, 152). Recent studies in rodents (153) have confirmed an important role for AMPA receptors that are located on pre-OLs and mediate their death, as opposed to immature OLs found in the study of Follett and colleagues (96). Hence, this study was important since suggested an additional possible pathophysiological mechanism for the HI-related injury and illustrated a specific vulnerability of immature OLs (O4+/O1+) compared to the younger (O4+/O1) and older OL cells (MBP+). However, this study is not without flaws and further validation of this model is required.

To study possible interactions between HI and infection/inflammation during the perinatal period, a new rodent model was introduced in 2001 (84). A single dose of LPS was given to 7-day-old rat pups 4 h before unilateral occlusion which was implemented as previously described (93). The combination of LPS with HI induced a more severe brain injury compared to HI alone. For instance, areas of infarction were enlarged, and there were increased concentrations of astrocytes and microglia around infarction zones. Mechanisms of immune system involvement in tissue damage were explored, and alterations in TLRs, and CD14 protein concentrations were found. Further research is needed to elucidate the pathways of interaction of pathophysiological processes.

In subsequent years, two further models of BCAO were reported, in addition to the models of Uehara (PND5) and Jelinski (PND7). In the first model (144), surgery was performed in rat pups at PND1, and no hypoxia conditions were applied. Mild diffuse injury was similar to that seen in human infants with PVL. More specifically, the injury was restricted to white matter and characterized by decreased numbers of immature and mature OLs, and activation of macrophages and microglia (144). In the second new model, surgery was undertaken in rats at PND4, and, in addition to bilateral arterial occlusion, rats were exposed to 10–15 min of 8% oxygen hypoxic conditions. Severe gray and white matter damage occurred with necrotic areas, loss of mature OLs and thinning of the corpus callosum (146).

The three models of BCAO described above produced mild and severe brain damage, with selectively affected white matter or gray matter. The only BCAO model undertaken in PND1 rats (144) produced a selective diffuse white matter injury similar to that seen in most PVL cases. The extent of the damage may be related to the younger age, different anesthesia agent used, or to the method of artery occlusion. Anesthesia duration may affect the degree of the injury (154), which is not specified in the study reports.

In 2002, Back et al. published data demonstrating that late OL progenitors (O4+/O1−) are selectively vulnerable to HI (56), similar to late OL progenitors in human infants at 24–32 weeks of gestation (44). Those OL progenitors were found to predominate at PND 2 (56–58). At PND7, immature OLs comprise 92% of all OL cells in the corpus callosum and are significantly less sensitive to HI compared to late OL progenitors (56). These findings gave rise to subsequent work using rats and mice less than 1 week old.

HI performed in rat pups at PND3 with subsequent hypoxia for 10 min produced a mild injury of cortical areas, thalamus and basal ganglia, necrotic and apoptotic cells, and astrogliosis. However, white matter damage was not quantified (145). The authors were successful in producing consistent mild brain damage due to preserving HI parameters: duration of surgery was kept within 10 min, subsequent hypoxia performed with 6–7% oxygen, +35–37°C, and 98–100% humidity.

When the surgery was done at PND3 with subsequent hypoxia using 8% oxygen for 60 min, mild white and gray matter damage was observed (147). For the first time, gender differences in HI-associated pathophysiological processes were described: post-hypoxia injections of 2-iminobiotin (2-IB) prevented brain damage in females only by suppressing release of cytochrome *c* from mitochondria. Results indicated that HI-induced cell death is mediated by the caspase-3 pathway in females, but not in males. This study reported a high degree of consistency in their results likely due to keeping the duration of isoflurane-maintained surgery constant (154).

A mouse model that employed HI performed at PND5 was developed recently (148). Different durations of hypoxia were investigated, and 70 min of hypoxia was found to induce a pattern of diffuse white (MBP staining) and focal gray (MAP2) matter injury similar to that in preterm infants with PVL. Innate and adaptive immune responses were recorded, and implicated in the induction of white matter damage. While MBP staining is not sufficient alone to replicate human pathology, it can indicate pathology of mature OLs and partially reveal the pathological mechanisms.

## Advancements in Clinical Management of Perinatal Complications in Human Infants Based on Rodent HI Model Research

Rigorous research using HI models during the last decades have elucidated mechanisms of prenatal white and gray matter injury, and enabled the development of clinical recommendations to diminish injury consequences in premature infants. Avoidance of factors implicated in damage occurrence has led to introduction of following clinical approaches.

### Maintenance of Infant Cerebral Perfusion

Maintenance of infant cerebral perfusion was found to be important based on studies done using the rodent HI model (101, 155), and significant effort has been put into the development of methods to detect infants with impaired cerebrovascular regulation. Significant decrease in cerebral blood flow during the first 12 h of life, as detected by near-infrared spectroscopy (NIRS) and color Doppler ultrasonography (cD-USG), correlates with poor neurological outcome or death (156, 157). Consequently, an array of critical care methods aimed at hypotension management has been developed (158). Prior to birth, use of magnesium sulfate, which, in addition to its vasodilation properties, has antioxidant and anticytokine effects, was found to be beneficial in preventing brain injury and is currently used in clinical practice (135, 159–162).

### Post-Injury (PND7) Hypothermia

Post-injury (PND7) hypothermia (+31–34°C) in the HI model was found to reduce brain damage to the cortex (150), including hippocampus, thalamus, and basal ganglia (120), with improvement evident at PND42 (163). Similar results were found in fetal sheep (164) and newborn piglets (165), which have greater similarities to human brain maturation rates and structures than rodents (149, 166). Therapeutic hypothermia is now the standard of care for brain injury control in term infants with perinatal hypoxic–ischemic encephalopathy (167, 168); however, it is not yet recommended for preterm infants.

### Prevention of Pre-OL Death

Prevention of pre-OL death (92) and maturation arrest are of greatest value. Activation of glutamate NMDA (74, 169) and AMPA (96) receptors is associated with OL death in HI However, no clinical trials investigating the use of glutamate antagonists have been commenced. Activation of another glutamate receptor type, the mGluR, which is developmentally regulated and is maximally expressed on the most vulnerable pre-OLs (170), was found to counteract oxidative stress and protect OLs (171). Others have investigated the role of the constituents of natural health products, and sulforaphane, as potential therapies to reducing the effects of oxidative stress and inflammation, in the prevention of CP (172). These promising findings are still awaiting translation to clinic.

Measures to address infection/inflammation prevention, identification, and management during pregnancy have also been considered (173). Despite the fact that the link between gestational inflammation and brain damage was not confirmed in several studies (80, 81), at present the standard of care in the US involves the administration of broad spectrum antibiotics when chorioamnionitis is diagnosed (174).

## Discussion: Limitations of Rodent HI Models and Their Clinical Applicability

The focus of the present paper is on rodent HI models since rodents are the most cost-effective and the most used species in laboratory research. Furthermore, availability of antibodies and transgenic animals allows comprehensive exploration of many physiological mechanisms. However, considerable limitations of rodent models restrict their use, and the present review is intended to reveal gaps in the accumulated knowledge and to lessen redundancies in ongoing research.

### Differences in Brain Organization and the Time Line of Brain Maturation in Rodents and Humans

Research undertaken in the HI rodent models during the last decades has found many similarities between the mechanisms of brain injury and damage evolution in rodents and human infants with CP. Fine-tuned HI models successfully reproduce hypoxic–ischemic conditions, the apoptotic–necrotic pattern of white and gray matter damage, and neuromotor impairments. However, the greatest limitation of the HI rodent models is the difference between rodents and humans in the overall complexity of brain organization, and the discrepancies in the rate of maturation in any time period (149).

Hypoxia–ischemia rodent models can be used to induce brain injury at a similar stage of *cellular* development: between 24 and 32 weeks of gestation in human, and at PND2-3 in rodents. Most of the oligodendroglial lineage cells are at the stage of pre-OLs at these time points, and these are maximally vulnerable to hypoxic–ischemic injury (Table 1). However, despite the existence of a similar cellular stage of development, the speed and duration of *structural* maturation of the developing brain is significantly different in rodents and humans. There is a huge difference between the size of a human and a rodent brain and, in particular, the cerebral cortex, which is associated with considerably longer neurogenesis in humans (175, 176). Accordingly, the structure and relative size of the subplate, which is the major target of hypoxic–ischemic injury, is different in humans and rodents. In humans, the subplate is a more dynamic structure, is more complex and compartmentalized, and is involved to a greater extent in the formation of the cortex pathways than in rodents (177, 178). In rodents, the subplate is a complete structure by E16–18 (179), while in humans it reaches its maximal thickness considerably later, at 24–32 weeks of gestation (37). Moreover, the increased complexity of the human subplate may contribute to differential vulnerability in response to HI in rodents and humans (180).

In an optimized HI rodent model, injury is induced at PND2–3, to match the developmental stage of the OLs to that in humans (24–32 weeks of gestation) (55–57, 96). Moreover, striking similarities between reduced cortical activity following HI and subsequent brain development both in infants and in rodents have been found (33). However, the state of subplate development is different at these time points: at PND2–3 in rodents, the subplate is already losing cells and function (181), whereas in humans at 24–32 weeks of gestation the subplate is at its maximal thickness and is actively participating in corticogenesis (37). Therefore, the consequences of subplate damage will be significantly different between humans and rodents, which indicates the limited clinical relevance of this HI rodent model (180).

### Sex Differences in Response to Prenatal Injury

Another important limitation of HI models concerns sex differences relating to brain vulnerability and the ability to repair in humans and rodents. In low weight infant boys, the incidence of intraventricular hemorrhage is significantly higher (182), as is the incidence of spastic CP, compared to girls (by 20–30%) (183–186). Moreover, the recovery of brain volumes is lower in boys than in girls: low weight preterm boys still present with white matter deficits at the age of 8, while matched-weight girls do not (187).

In the rodent model, sex differences in the pathophysiological mechanisms underlying brain damage evolution and recovery were observed. In females, caspase-dependent apoptosis has been described (147, 188), while in males, cell death and subsequent brain damage is associated with a different pathway involving PARP activation (189). Hypothermia following HI injury was beneficial for females only (163); however, the finding of sex differences was not a primary aim of the study, and the results, therefore, require additional verification, as acknowledged by the authors. Despite the different sex physiological processes in response to injury, the final extent of brain damage following HI at PND7 (190, 191) or before puberty (192), was similar in males and females. However, the above studies were done in PND7 and older animals, when development of a rodent brain roughly corresponds to a term human infant. Delivery of acute HI injury at PND3 (193) or chronic HI between PND3 and PND11 (194) induced significantly greater damage of white and gray matter, greater reduction in brain volume and more cognitive deficits in males compared to females, thereby resembling the damage pattern observed in preterm infants.

In rodents and humans, sex-specific responses to hypoxic–ischemic injury are different, making the rodent model a non-ideal choice to study gender-dependent mechanisms of CP. However, sex-specific influences are complex, and the contradictory evidence (193, 194) warrants further research to elucidate sex-specific mechanisms of HI-response maturation.

### Modeling Motor Impairments

Spastic CP is characterized by aberrant reflex development and the lack of voluntary control (8). Unilateral damage of the CST during the prenatal period, when motor networks are at the stage of refinement – strengthening useful and eliminating redundant connections – results in ipsilateral aberrant CST projections and abnormal reflexes that manifest in spasticity (195, 196).

Considerable data have been accumulated regarding motor impairments in the rodent HI model. Despite rodents not showing the severe spastic impairments observed in human patients, gross motor function impairments could be said to resemble those in hemi- or diplegic CP (146, 190, 197–200). Most studies were undertaken within a week of the insult, and a relatively small number of papers have addressed the correlation between specific lesions and motor performance in the long term (2). Impairments in fine skilled voluntary movement following HI are studied even more rarely (198), even though they are present in most cases of spastic CP due to corticospinal pathway damage (201–204).

There are several concerns regarding the modeling of CST injury in rodent HI models. First, the overall anatomy of the CST differs considerably from that in humans. Rodents have very few direct connections between the motor cortex and motor neurons in the ventral horn of the spinal cord (205, 206), specifically for motor neurons innervating the muscles of the fingers. Therefore, impairments of skilled voluntary movements may be less pronounced in rodents, and require sensitive behavioral tests for identification.

Another important anatomical difference is the number of axons within the CST, which decussate in the medulla to the contralateral side. In rodents 2–4% axons remain uncrossed (207), whereas in humans about 8–15% of axons stay at the ipsilateral side (195). In humans, there is an even greater amount of ipsilateral axons within the CST in the prenatal period when injury occurs. These form transient connections with spinal cord neurons and disappear during postnatal development due to the functional competition with contralateral projections. In patients with hemiplegia, those ipsilateral connections are preserved and contribute to spastic symptoms (196, 208, 209). In rodents, research suggests that the mostly contralateral pattern of innervation is determined from the onset with no transient ipsilateral projections comparable to those in humans (210, 211). However, in rodents, if brain lesions are induced in the first week after birth, when the majority of corticospinal fibers are growing into the spinal cord, considerably enlarged ipsilateral connections are formed that might resemble the aberrant ipsilateral CST projections observed in CP patients (212, 213). Indeed, mice demonstrated mirror voluntary bilateral synchronous movements following CST injury at PND0-3 that resembled mirror movements in CP patients (214).

In addition to governing voluntary fine movements of the forelimb, the CST plays an important role in the fine-tuning of spinal reflexes during spinal networks maturation, both in rodents (215, 216) and humans (211, 217, 218). However, the extent of that influence is likely different in rodents and humans, as evidenced by the effect of prenatal CST damage. More specifically, in humans this type of damage is associated with aberrant reflexes and disabling spasticity, while in rodents the effect is considerably weaker.

In summary, the anatomy of the CST is considerably different in rodents and humans, which limits clinical applicability of HI models. However, there is still a possibility of mimicking and studying specific processes involved in functional alteration and restoration, which may contribute to the development of treatment strategies in humans. For example, finding the crucial role of CST influence on fine-tuning of spinal modules during their maturation suggests that CP infants would benefit from boosting activity in the residual corticospinal innervation that might restore spinal connectivity pattern and reduce spasticity in future (211). In the rodent model, stimulating the HI-injured hemisphere will have an overall similar effect, though less pronounced in the ipsilateral side, due to less CST ipsilateral connections in rodents compared to humans.

### The Rodent Model Versus Large Animal Models

The rodent HI model is the most convenient, cost-effective, and widely used animal model and has yielded a substantial body of knowledge on CP pathophysiology. However, limitations of the rodent HI model have prompted research in large animal species with greater similarity to humans. The fetal instrumented sheep model has been characterized: preterm fetal fetus (95 days of gestation, equivalent to 24–28 gestational weeks in humans) was found to display cerebral hemodynamics similar to that in the human fetus, both in normal conditions (219, 220) and in response to HI (221, 222). Moreover, the gyrencephalic structure of the sheep brain cerebrum and the major stages of neurodevelopment are similar to those in humans (223–225). In addition, the larger size of sheep allows chronic monitoring of the fetus and relevant clinical interventions have been developed based on that model (164). The major disadvantage of the instrumented fetal sheep model is the lack of opportunity to study motor deficiencies, especially dexterity movements, associated with hypoxic–ischemic insult (226).

A non-human primate model developed in baboons at 125 days of gestation, equivalent to 26 weeks of gestation in human, has shown similarities with human preterm infants in the pattern of white matter injury (227). The primate model is associated with the necessity for artificial ventilation (228) that may resemble a specific human condition, though overall it may restrict the interpretation and applicability of the results.

A HI model in a newborn piglet has demonstrated electrophysiological and neuropathological disturbances similar to those in the asphyxiated term human infant (229). The model is able to mimic comparable changes in brain white matter to a human newborn (230). However, high mortality and discrepancies in protocols result in variability in the extent of brain injury and make it difficult to draw firm conclusions.

### Intra- Versus Extra-Uterine Models

Differences in the rate of brain maturation between rodents and humans prevent concomitant times when both structural and cellular components are at the same level of development. Thus, in *intra-uterine studies* in rodents (E17–19), injury is induced when the brain subplate is already a complete structure (179). This period corresponds to 24–32 gestational weeks in humans when the subplate reaches its maximal thickness (37) and synaptogenesis begins both in rodents (231, 232) and in humans (233, 234). While at this stage the level of subplate development is comparable in both rodents and humans, the subsequent differentiation and function of the subplate in humans is considerably different from that in rodents (180). Moreover, maximal population of pre-OLs in rodents is found between PND2 and PND5, while in humans the peak of subplate development is between 23 and 32 weeks of gestation that coincides with the period of highest vulnerability to perinatal brain injury (32).

By contrast, extra-uterine HI models aim to induce brain injury during maximal OL vulnerability, for example, between PND2 and PND5 in rodents. Therefore, intra-uterine rodent models target different aspects of brain pathophysiology associated with HI (65). Intra-uterine growth restriction models are being developed in guinea pigs (235–237), mice (238), rabbits (239), and rats (67, 172, 240, 241).

The instrumented fetal sheep model makes it possible to induce hypoxic–ischemic injury at the stage of both the subplate and pre-OLs being at the similar stages of development in sheep and humans and, therefore, more closely mimics events accompanying hypoxic–ischemic insult (226).

## Conclusion

The rodent HI model continues to contribute to CP research. It mimics many pathophysiological changes in human patients with spastic CP and allows the study of the mechanisms underlying pre- or perinatal brain injury. Unilateral injury induced at PND3-4 in combination with precisely adjusted temperature and oxygen level and surgery duration can reproduce brain damage patterns and motor impairments similar to those in hemiplegic CP patients. Moreover, the model allows investigation of new methods of brain damage prevention and repair both at the cellular (introduction of pharmacological or biotechnological agents interrupting the cascade of pathophysiological events) and the systemic level (stem cell transplantation or behavioral manipulations stimulating anatomical and functional restoration).

Research done using the HI rodent model has identified factors implicated in brain pathophysiology, and has made important contributions to the current clinical practice of treating CP patients. For instance, postnatal hypothermia, management of decreased cerebral blood flow in newborns, and management of prenatal infection are common standards of care in many countries at present. However, the model has important limitations, such as overall anatomical differences between human and rodent brains, discrepancies in the rate of maturation at any time period, and in gender-specific mechanisms. These limitations restrict the clinical applicability of the results. The rodent model is an ideal cost-effective model for proof-of-concept studies and investigation of molecular mechanisms that can be validated subsequently in large pre-clinical animal models, such as the instrumented fetal sheep (226), and the monkey model that, in addition to physiological and size similarities, allows evaluation of functional outcomes.

## Method

The goal of the present study was to identify HI rodent models developed to date, analyze their contributions to the understanding of CP pathophysiological mechanisms, and to comment on the overall potential of the HI rodent model to mimic those mechanisms. A systematic literature search was conducted in May–July 2015 using PubMed, Scopus, Medline, Cochrane Library, and Web of Science databases in English. The following search strategies were used.

### PubMed (All Years)

Title/Abstract (“hypoxia ischemia” OR “hypoxic ischemic” OR “BCAO”) AND Title/Abstract (“rat” OR “rats” OR “mice” OR “mouse”) AND Title/Abstract (“cerebral palsy” OR “white matter” OR “injury”) returned 1434 results.

### Scopus

Title, abstract, keywords (“hypoxia ischemia” OR “hypoxic ischemic” OR BCAO) AND Title, abstract, keywords (“rat?” OR “mice” OR “mouse”) AND Title, abstract, keywords (“cerebral palsy” OR “white matter” OR “injury”) returned 1364 results.

### Medline (OVID MedLine without Revisions, 1996 – July week 2, 2015)

Title/Abstract (“hypoxia ischemia” OR “hypoxic ischemic”) AND Title/Abstact (“rat” OR “rats” OR “mice” OR “mouse”) AND Title/Abstact (“cerebral palsy” OR “white matter” OR “injury”) returned 1554 results.

### Cochrane Library (All Databases, All Years)

Title, abstract, keywords (“hypoxia ischemia” OR “hypoxic ischemic” OR BCAO) AND (rat OR rats OR mice OR mouse) AND (“cerebral palsy” OR “white matter” OR injury) returned 3 results.

### Web of Science (without Restrictions, All Years)

Title: (“hypoxia ischemia” OR “hypoxic ischemic” OR BCAO) AND Topic: (rat OR rats OR mice OR mouse) AND Topic: (“cerebral palsy” OR “white matter” OR injury) returned 1341 results.

Titles and abstracts of found papers were screened to determine if they fell into the scope of the review. From the selected papers, only those which satisfied the following criteria were analyzed.

### Criteria for Paper Inclusion into Analysis

The following papers were included: papers that were relevant to the subject, clearly described methods of research, had control groups, used appropriate outcome measures in which subjects are randomized and data scoring were blinded, and that report limitations of the research conducted. A total of 11 distinct rodent HI and two ischemic models (Table 3) were identified and analyzed in our review.

## Author Contributions

PR – drafting of manuscript, revision, and editing. TB – drafting of manuscript, revision, and editing. SM – edits and revisions to manuscript. JY – edits and revisions to manuscript. MF – concept of review, editing, revision, and final approval of manuscript.

## Conflict of Interest Statement

The authors declare that the research was conducted in the absence of any commercial or financial relationships that could be construed as a potential conflict of interest.
